# Mean platelet volume as a biomarker for liver fibrosis in patients with Non-alcoholic fatty liver disease

**DOI:** 10.1371/journal.pone.0318847

**Published:** 2025-02-18

**Authors:** Eun Hye Cho, Jee Ah Kim, Min-Seung Park, Min-Jung Kwon, Hyosoon Park, Hee-Yeon Woo

**Affiliations:** Department of Laboratory Medicine, Kangbuk Samsung Hospital, Sungkyunkwan University School of Medicine, Seoul, Korea; Kaohsiung Medical University, TAIWAN

## Abstract

**Introduction:**

Non-alcoholic fatty liver disease (NAFLD) is the most prevalent cause of chronic liver disease worldwide. Accurate assessment of liver fibrosis is essential for the prognosis and management of NAFLD. Mean platelet volume (MPV), an index of platelet activation, has been associated with liver fibrosis in various chronic liver diseases; however, its role in NAFLD remains uncertain.

**Methods:**

This study analyzed data from 70,830 patients with NAFLD who underwent a comprehensive health examination between 2015 and 2019. Patients were stratified into three groups based on the fibrosis-4 (FIB-4) index: low (<1.30), indeterminate (1.30–2.67), and high (>2.67). We evaluated the differences in MPV among these groups and analyzed the association between MPV and the FIB-4 index.

**Results:**

MPV varied significantly across the three groups categorized by the FIB-4 index (p < 0.001). Additionally, a weak but statistically significant positive correlation was observed between MPV and the FIB-4 index (rho =  0.170, p <  0.001). Multivariable linear regression adjusted for multiple covariates showed that MPV remained significantly associated with the FIB-4 index (β =  0.01, p <  0.001).

**Conclusions:**

MPV may serve as a supplementary non-invasive marker for assessing liver fibrosis in patients with NAFLD. Further studies are required to validate these findings and explore the utility of MPV in conjunction with other non-invasive markers.

## Introduction

Non-alcoholic fatty liver disease (NAFLD) is the most prevalent cause of chronic liver disease, with a global prevalence of 30% [1]. It encompasses a spectrum of conditions, ranging from simple steatosis to non-alcoholic steatohepatitis, fibrosis, and cirrhosis [2]. Given that NAFLD can progress to hepatocellular carcinoma, the assessment of liver fibrosis in patients with NAFLD is critical for determining prognosis and directing management strategies.

Liver biopsy has long been regarded as the gold standard for evaluating liver fibrosis [3]. However, its invasiveness, associated costs, and potential complications have prompted the development of various non-invasive markers. Among these, the fibrosis-4 (FIB-4) index, which includes age, aspartate aminotransferase, alanine aminotransferase, and platelet count, has been extensively validated and is frequently utilized in clinical practice to estimate the degree of liver fibrosis [4,5].

Mean platelet volume (MPV) is a hematologic parameter that reflects platelet size and serves as an index of platelet activation [6]. Previous studies involving patients with hepatitis B and C have demonstrated that increased MPV is associated with liver fibrosis [7–9]. However, the relationship between MPV and the degree of liver fibrosis in individuals with NAFLD remains uncertain. Notably, only one study has indicated that MPV does not correlate with fibrosis in patients with NAFLD [10].

In this study, we aimed to investigate the association between MPV and liver fibrosis in patients with NAFLD, utilizing the FIB-4 index to assess the potential of MPV as a biomarker for evaluating liver fibrosis. Understanding this relationship could enhance the utility of MPV as a non-invasive marker for the stratification and management of patients with NAFLD.

## Methods

### Study population

The Kangbuk Samsung Health Study is a cohort study involving Korean men and women who underwent a comprehensive annual or biennial health examination at Kangbuk Samsung Hospital Total Healthcare Centers in South Korea [11]. After removing duplicates, data from 301,899 participants who underwent a comprehensive health examination between 2015 and 2019 were included in this study. Initially, 18,740 participants were excluded due to testing positive for hepatitis B surface antigen or hepatitis C antibody, or due to a lack of test results. Subsequently, 1,152 participants with a history of hepatocellular carcinoma or with an unknown history were excluded. To eliminate potential cases of alcohol-induced liver disease, 54,500 participants were excluded if they were women with an alcohol consumption of ≥ 20 g/d, men with an alcohol consumption of ≥ 30 g/d [12], or if they did not respond to questions regarding alcohol consumption. Additionally, 156,556 participants whose abdominal ultrasounds did not indicate fatty liver or who did not undergo an abdominal ultrasound were excluded. Finally, 25 participants with missing data required for FIB-4 score calculation and 50 participants without MPV data, and 46 participants with missing values for any of the covariates included in the multivariable regression analysis were excluded from the analysis. After these exclusions, 70,830 patients with NAFLD were included in the final analysis. This study was approved by the Kangbuk Samsung Hospital Institutional Review Board (approval number: 2023-07-059). Given the retrospective nature of the study and the use of anonymized data, the requirement for informed consent was waived. The data used in this study was accessed on August 3, 2023.

### Measurements

Alcohol consumption was assessed through a self-reported questionnaire that inquired about the frequency and amounts of alcoholic beverages consumed per drinking day.

Platelet counts and MPV were measured using the Sysmex XE-2100D automated hematology analysis system (Sysmex Corporation, Kobe, Japan). Aspartate aminotransferase and alanine aminotransferase levels were measured using the Cobas c702 analyzer (Roche Diagnostics, Tokyo, Japan). The FIB-4 index was calculated using the following formula: FIB-4 =  (age [years] ×  AST [U/L])/ (platelet count [×10^9^/L] ×  ALT [U/L]^1/2^) [4]. The cutoff values for the FIB-4 index are < 1.30 for the absence of advanced fibrosis and > 2.67 for the presence of advanced fibrosis [5].

Abdominal ultrasonography was performed by experienced radiologists who were blinded to the objective of the study. Fatty liver was diagnosed according to standard criteria [13,14].

### Statistical analysis

Categorical variables are presented as counts and percentages, while continuous variables are expressed as medians and interquartile ranges. Differences in variables among groups categorized by the FIB-4 index were analyzed using the Kruskal–Wallis test, followed by a post-hoc Tukey’s test for multiple comparisons. Spearman correlation analysis was employed to assess the relationship between MPV and the FIB-4 index. Multivariable linear regression analysis was performed using the FIB-4 index as the dependent variable. MPV was included as the main independent variable, and the following covariates were adjusted for in the model: age, body mass index (BMI), diabetes mellitus (DM), albumin, AST, ALT, and platelet count. All independent variables were checked for multicollinearity using the variance inflation factor (VIF), with a cutoff of 10 indicating high multicollinearity. All statistical analyses were performed using IBM SPSS software version 28.0 (IBM Corp., Armonk, NY, USA). A p-value of < 0.05 was considered statistically significant.

## Results

**Fig 1:**
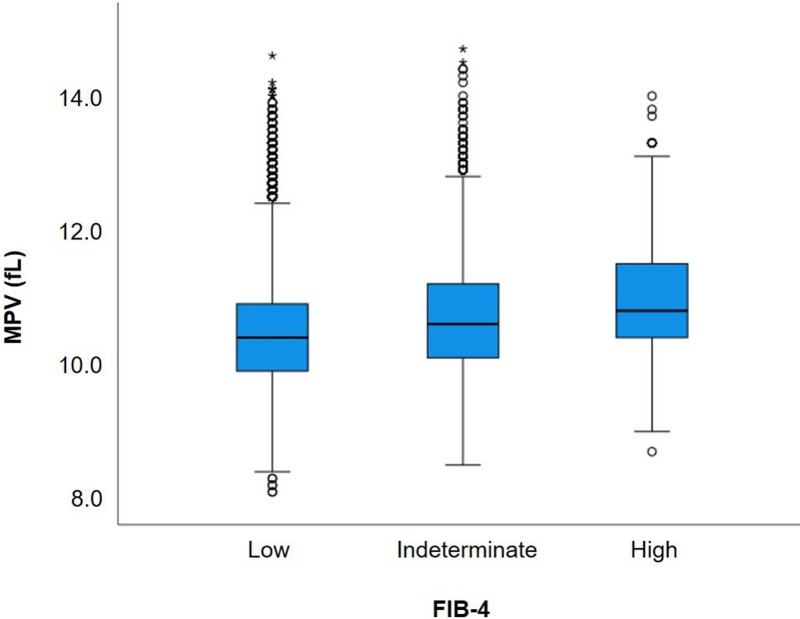
Distribution of MPV across the FIB-4 index categories in patients with NAFLD. MPV, mean platelet volume; FIB-4, fibrosis-4; NAFLD, non-alcoholic fatty liver disease.

Study participants were categorized into three groups based on the FIB-4 index: low (<1.30), indeterminate (1.30–2.67), and high (>2.67). The proportions of patients in the low, indeterminate, and high FIB-4 index groups were 91.9% (65,070/70,830), 7.7% (5,441/70,830), and 0.5% (319/70,830), respectively. The characteristics of the participants are summarized in Table 1. All examined variables showed statistically significant differences among the three groups (p < 0.001), except for BMI, which showed no significant difference between the low and high FIB-4 index groups (p = 0.694) (Table 2). The median MPV for the low, indeterminate, and high FIB-4 index groups were 10.4 fL, 10.6 fL, and 10.9 fL, respectively (Fig 1). Spearman correlation analysis indicated a statistically significant, albeit weak, positive correlation between MPV and the FIB-4 index (rho =  0.170, p < 0.001). On multivariable linear regression adjusted for age, BMI, DM, albumin, AST, ALT, and platelet count, MPV remained significantly associated with FIB-4 index (β =  0.01, p <  0.001) (Table 3).

**Table 1:** Characteristics of the study population.

Variables	Value
Age (y)	42 (36–48)
Sex	
Male, n (%)	55,201 (77.9)
Female, n (%)	15,629 (22.1)
AST (IU/L)	23 (19–30)
ALT (IU/L)	29 (21–44)
Albumin (g/dL)	4.8 (4.6-5.0)
BMI (kg/m²)	26.0 (24.2-28.1)
DM	
Yes	7817 (11.0%)
No	63013 (89.0%)
Platelet count ( × 10^9^/L)	257 (224–294)
FIB-4 index	0.70 (0.54–0.93)
Low, n (%)	65,070 (91.9)
Indeterminate, n (%)	5,441 (7.7)
High, n %)	319 (0.5)
MPV (fL)	10.4 (9.9–10.9)

Continuous variables are presented as medians and interquartile ranges and categorical variables are presented as counts and percentages.

AST, aspartate aminotransferase; ALT, alanine aminotransferase; BMI, body mass index; DM, diabetes mellitus; FIB-4, fibrosis-4; MPV, mean platelet volume.

**Table 2:** Comparison of variables among groups categorized by the FIB-4 index.

	Low	Indeterminate	High	p-value
MPV	10.4 (9.9–10.9)	10.6 (10.1–11.2)	10.8 (10.4–11.5)	<0.001
Age	41 (36–47)	57 (50–64)	59 (47–67)	<0.001
BMI	26.0 (24.2–28.2)	25.7 (24.0–27.6)	26.0 (24.4–28.6)	<0.001^a^
DM	6301 (9.7%)	1404 (25.8%)	112 (35.1%)	<0.001
AST	22 (18–29)	30 (24–44)	68 (40–125)	<0.001
ALT	29 (20–43)	30 (21–49)	48 (30–82)	<0.001
Albumin	4.8 (4.6-5.0)	4.7 (4.6-4.9)	4.7 (4.5-4.9)	<0.001
Platelet	261 (229–297)	206 (181–234)	172 (140–214)	<0.001

^a^p  =  0.694 for the comparison of BMI between the low and high FIB-4 index groups.

Continuous variables are presented as medians and interquartile ranges and categorical variable is presented as count and percentage.

FIB-4, fibrosis-4; MPV, mean platelet volume; BMI, body mass index; DM, diabetes mellitus; AST, aspartate aminotransferase; ALT, alanine aminotransferase.

**Table 3:** Multivariable linear regression analysis of the association between MPV and FIB-4 index with adjustment for covariates.

Variable	Estimates (β)	p-value	VIF
MPV	0.010	<0.001	1.163
Age	0.022	<0.001	1.173
BMI	0.002	<0.001	1.114
DM	0.008	<0.001	1.085
AST	0.020	<0.001	3.032
ALT	-0.007	<0.001	3.209
Albumin	0.0008	0.770	1.115
Platelet count	-0.003	<0.001	1.187

MPV, mean platelet volume; FIB-4, fibrosis-4; BMI, body mass index; DM, diabetes mellitus; AST, aspartate aminotransferase; ALT, alanine aminotransferase.

## Discussion

The relationship between MPV and FIB-4 index in patients with NAFLD was investigated in the present study. Our findings revealed significant differences in MPV values across the three FIB-4 index categories. Additionally, a weak but statistically significant positive correlation was identified between MPV and the FIB-4 index. These results underscore the potential utility of MPV as a complementary marker for assessing fibrosis severity in patients with NAFLD.

The differentiation of hepatic stellate cells into myofibroblasts represents a crucial mechanism in the development of liver fibrosis. In this process, platelets play a significant role by releasing profibrotic cytokines such as transforming growth factor-beta and platelet-derived growth factor-beta [15,16]. Transforming growth factor-beta stimulates the activation and differentiation of hepatic stellate cells into myofibroblasts, resulting in excessive extracellular matrix deposition [15]. Platelet-derived growth factor-beta acts as a mitogen and chemotactic factor for activated hepatic stellate cells and myofibroblasts, thereby promoting fibrosis [16,17]. The synergistic effect of these cytokines accelerates the progression of liver fibrosis.

MPV serves as an indicator of platelet size and activity, with larger platelets typically reflecting increased systemic inflammation and platelet activation [6]. In the context of NAFLD, where chronic inflammation drives disease progression [18], increased MPV may signify more severe liver injury and fibrosis. According to a previous study by Miele et al. [19], pro-inflammatory changes in platelets have been observed in patients with NAFLD, further supporting this relationship. Additionally, prior research has demonstrated an association between MPV and the severity of fibrosis in chronic liver diseases such as hepatitis B and C [7–9]. In patients with hepatitis B, elevated MPV was associated with advanced fibrosis, revealing a significant difference between mild and advanced fibrosis categories. Similarly, in patients with hepatitis C, MPV was significantly higher in those with advanced fibrosis, thereby reinforcing the potential of MPV as a non-invasive marker of fibrosis progression.

Conversely, our study specifically focused on patients with NAFLD and analyzed the MPV according to FIB-4 index categories. Our findings revealed that patients classified within the high FIB-4 index category exhibited higher MPV compared to those in the low and indeterminate FIB-4 index categories. This observation suggests that MPV may be associated with the severity of liver fibrosis in NAFLD and aligns with the understanding that platelet activation contributes to the pathogenesis of fibrosis. The significant differences in MPV among the FIB-4 categories highlight the potential role of MPV in identifying patients at higher risk for advanced fibrosis in the context of NAFLD. Furthermore, MPV may serve as an early indicator of disease progression or treatment response, facilitating more effective management of NAFLD.

This study demonstrated a weak yet statistically significant positive correlation between MPV and the FIB-4 index. This finding aligns with the established understanding that increased platelet activation plays a role in fibrosis progression [15]. However, the weak correlation indicates that MPV alone is not a robust predictor of fibrosis severity in individuals with NAFLD and may have limited utility as a standalone marker for risk stratification in clinical practice. Despite these limitations, MPV remained independently associated with the FIB-4 index after adjusting for covariates, including age, BMI, DM, albumin, AST, ALT, and platelet count. This finding highlights the potential role of MPV as a complementary marker for fibrosis assessment. Although MPV alone may not significantly improve the identification of advanced fibrosis, its inclusion in a composite scoring system could provide incremental value in improving diagnostic accuracy. Further studies incorporating MPV alongside other noninvasive fibrosis markers are needed to improve the accuracy of fibrosis risk stratification and reduce the dependence on liver biopsy.

A previous study revealed a moderate correlation between MPV and FibroScan results, yielding a correlation coefficient of 0.476 [7]. FibroScan provides a direct measurement of liver stiffness, which may offer a more accurate reflection of fibrosis severity compared to the FIB-4 index [20]. The stronger correlation between MPV and FibroScan indicates that MPV may be more closely associated with the physical alterations linked to fibrosis, as opposed to the indirect markers utilized in the FIB-4 index. This observation suggests that increased MPV is more directly related to these alterations, highlighting its potential as a valuable marker for assessing fibrosis severity.

This study has some limitations. First, its cross-sectional design limits our ability to establish causal relationships between elevated MPV and fibrosis progression. Longitudinal studies are necessary to ascertain whether changes in MPV over time are directly correlated with disease progression. Second, fibrosis assessment was conducted using the FIB-4 index rather than liver biopsy or FibroScan, owing to the reliance on health examination data where liver biopsy and FibroScan were not feasible. Third, variability existed in the sizes of groups categorized by the FIB-4 index.

In conclusion, our study illustrates that MPV is significantly associated with the severity of liver fibrosis in patients with NAFLD. These findings underscore the potential of MPV as a valuable, non-invasive marker for assessing fibrosis risk and guiding the management of patients with NAFLD. As a non-invasive and easily measurable parameter, MPV could be integrated into routine clinical practice to enhance the evaluation of fibrosis severity in this patient population.
